# Double balloon catheters: A promising tool for induction of labor in multiparous women with unfavorable cervices

**DOI:** 10.4274/jtgga.galenos.2018.2018.0084

**Published:** 2019-11-28

**Authors:** Fırat Tülek, Ali Gemici, Feride Söylemez

**Affiliations:** 1 Department of Obstetrics and Gynecology, Ankara University School of Medicine, Ankara, Turkey; 2 Department of Perinatology, Ankara University School of Medicine, Ankara, Turkey

**Keywords:** Double-balloon catheter, labor induction, unfavorable cervix

## Abstract

**Objective:**

To compare the effectiveness and safety of oxytocin and a cervical ripening balloon in women with unfavorable cervices for inducing labor.

**Material and Methods:**

A total of eighty pregnant women between 37-41 gestational weeks having singleton pregnancies and intact membranes with unfavorable cervices were randomized into two groups, cervical ripening balloon (n=40) and oxytocin infusion (n=40). The primary outcomes were the labor time and the route of delivery. Secondary outcomes were the effect of parity on time of labor, and obstetric and perinatal outcomes.

**Results:**

The median time to delivery was 9.45 hours in cervical ripening balloon group and 13.2 hours in the oxytocin group in multiparous women. The differences were statistically significant (p<0.001). The median time until delivery was 11.48 hours in cervical ripening balloon group and 13.46 hours in the oxytocin group; the differences were statistically significant (p<0.001). Cesarean delivery ratios were similar in both groups (p=0.431).

**Conclusion:**

The results of the present study are promising for balloon use, especially in multiparous women. It is beneficial to support these data with wide ranging population-based studies.

## Introduction

The mechanisms of birth and the initiating factors of labor are still obscure. The main goal of obstetrics is the health of both the mother and the baby. The management of the labor process is an important part of obstetric practice. Although labor initiates spontaneously in a vast majority of pregnancies, induction of labor (IOL) may be needed in a wide variety of conditions including post-term pregnancies, preterm premature membrane rupture, preeclampsia, eclampsia, hemolysis, elevated liver enzyme levels, and low platelet levels-HELLP syndrome, fetal demise, maternal diabetes mellitus, fetal distress, maternal cholestasis, and chorioamnionitis. In such situations, an unfavorable uterine cervix is one of the major obstacles to successful IOL (1).

The uterine cervix retains its physical integrity by remaining firm during pregnancy until the beginning of labor. Just before labor, the cervix softens and becomes more distensible; this process is called cervical ripening. However, if induction is indicated before onset of spontaneous labor, cervical conditions come to the fore for a successful delivery. The Bishop scoring system is commonly used for evaluation of the cervix (2,3). In general, when the Bishop score is less than six, the success rate of IOL is poor (3,4,5). Unfortunately, nearly 50% of women with an indication of IOL have an unfavorable cervix with a Bishop score of less than six (2).

Oxytocin is one of the commonly preferred pharmacologic methods used for inducing labor (6). However, systemic adverse effects including tachycardia, flushing, and uterine tachysystole are the disadvantages experienced during its infusion. The primary effect of oxytocin is on myometrial contractility and the secondary effect is on cervical ripening in interactions with steroid hormones, prostaglandins (PGs), and probably nitrite oxide (NO) (7,8).

Alternatively, a cervical ripening balloon is used for IOL. Such balloons may be speculated to cause fewer systemic adverse effects because of their local and limited effect and because they are mechanical in nature (9). On the other hand, high cost and application difficulties are some of the disadvantages experienced when these devices are preferred for IOL. The proposed mechanism of the cervical ripening balloon during labor induction is apparently mechanical, but interestingly, cervical ripening is also achieved pharmacologically via the Ferguson reflex. This is a neuroendocrine reflex and has as its afferent limb the sensory nerves from the vagina and cervix (Franken Hauser nerves), the ascending somatosensory pathways in the spinal cord (the anterolateral columns) and an incompletely described projection through the brainstem and medial forebrain bundle that ultimately reaches the hypothalamic magnocellular nuclei. The efferent limb of the reflex is the blood-borne carriage from the paraventricular nucleus and supraoptic nucleus of oxytocin. Oxytocin secretion is a consequence of this neuroendocrine reflex induced by tactile stimulation of the reproductive tract (9).

Although both procedures are implemented in different ways, both oxytocin and cervical ripening balloons have similar mechanisms in terms of their molecular action (8,9). Hypothetically, a cervical ripening balloon might have lesser adverse effects during IOL as compared with oxytocin because of having a closer physiologic mechanism to oxytocin secretion (10,11). In this study, we aimed to compare the effectiveness and safety of oxytocin (Synpitan^®^) and cervical ripening balloons (Cook^®^) for IOL in unfavorable cervices.

## Material and Methods

This prospective randomized study was conducted at a tertiary care hospital. Ethics committee approval was obtained (approval number 154-4958/2009). After giving informed consent, patients were assigned to one of the induction methods according to a computer-generated randomized list. A total of 80 pregnant women with singleton pregnancies between 37 and 41 gestational weeks and intact membranes with unfavorable cervices were randomized and assigned to either the cervical ripening balloon group (n=40) or the oxytocin infusion (n=40) group. A visual analogue scale (VAS) was used to determine pain perception in both groups. All participants rated the pain using the VAS during the labor process (in the VAS scale, pain is rated from zero to ten in which zero indicates no pain and ten the worst possible pain).

The presence of vaginal bleeding, pregnancies from in vitro fertilization, non-cephalic presentation, multi-fetal pregnancies, prior uterine or cesarean surgery, an estimated fetal weight over 4500 grams, and obstetric complications (hypertensive disorders, post-term pregnancies or fetal growth disorders) were the exclusion criteria for labor induction.

### Oxytocin infusion

Administration started with ten units of oxytocin per 1000 mL 0.9% saline with a rate of 10 mU/min adjusted by infusion pump. The infusion rate was increased by 1 mU/min every 15 minutes, until three strong uterine contractions were achieved in a one-minute period (200-250 Montevideo units). The maximum dosage was set as 60 mU/minutes. All cases were monitored for fetal heart rate and uterine contractions until delivery.

### Cervical ripening balloon insertion

All patients were placed in the lithotomy position. The balloon was inserted using ring forceps passing through the internal cervical ostium and into the extra-amniotic space. The uterine part of the balloon was inflated with 40 mL saline and pulled back to the level of the internal ostium and the vaginal part of the balloon was inflated with 20 mL saline. The position of the balloon was checked. Once the correct position was established, both balloons were inflated with up to 80 mL. All patients were monitored by means of fetal heart rate and uterine contractions until delivery.

The groups were compared with respect to the interval between the time of induction and delivery, the amnion membrane rupture time, pain perception during labor, the amount of hemorrhage after delivery, the route of delivery, and the first and fifth minute APGAR scores. The primary outcomes were the induction to delivery interval and the cesarean ratios. Secondary outcomes included the effect of parity on the time to the onset of labor, in addition to obstetric and perinatal outcomes.

### Statistical analysis

After performing normalization tests on parametric data, Student’s *t*-test was used for data with normal distribution. The Mann-Whitney U test was used for data with non-normal distribution. Non-parametric values were analyzed using the chi-square test. IBM statistics SPSS 20.0 software was used for statistical analysis. P values <0.005 were considered statistically significant.

## Results

The study was conducted at a tertiary care hospital in the department of obstetrics and gynecology. A total of 80 pregnant women aged between 22 and 29 years were enrolled. The mean ages were 24.75±3.57 years in the oxytocin group and 25.13±3.84 years in the balloon group. The demographic parameters of both groups were similar (Table 1). The cesarean ratio in the oxytocin group was 20% (eight out of 40 women) and in the balloon group it was 27.5% (11 out of 40 women). The cesarean ratios were similar between the groups (p=0.431) (Table 1). The mean time until delivery was 13.46 hours in the oxytocin group and 11.48 hours in the cervical ripening balloon group; the differences were statistically significant (p<0.001). In the multiparous women, the median time until delivery was 13.2 hours in the oxytocin group and 9.45 hours in the cervical ripening balloon group; the differences were statistically significant (p<0.001) (Table 2, 3). The estimated fetal weight and APGAR scores of the newborns were similar in both groups. The mean hemoglobin changes from the pre-partum to post-partum period were 1.7 g/dL; the hemoglobin changes were not statistically significant (p=0.884). Pain perception scores were higher in the oxytocin group and this change was statistically significant (p<0.001). Neonatal intensive care hospitalization was needed for 10% (four out of 40 babies) in the oxytocin group and 2.5% (one out of 40 babies) in the balloon group. The difference was not statistically significant (p=0.359). The mean time to amnion membrane rupture was 10.9 hours in the oxytocin group and 9.6 hours in the balloon group. This difference was statistically significant (p=0.019) (Table 4).

## Discussion

In this study, we compared the effects of oxytocin and cervical ripening balloons in term pregnancies with unfavorable cervices. The most commonly preferred induction agent is intravenous oxytocin infusion. Oxytocin is known as the hormone that starts uterine contractions. However, according to recent research, the effect of oxytocin for parturition is not limited to the initiation of uterine contractions, it is also effective in cervical ripening (8). The proposed mechanism of oxytocin for cervical ripening is the secretion of local NO and PGs (especially PG F2a) through oxytocin receptor action located at the amnion membrane and placental decidua (8). Similarly, cervical ripening balloons initiate secretion of oxytocin, NO and PGs (especially PG F2a) via the Ferguson reflex (9). At the molecular level, both induction methods may have similar mechanisms in IOL.

When comparing the two methods in respect to labor time, a shorter time interval from induction to delivery was observed in women induced with balloon catheters. In multiparous women induced with double-balloon catheters, the time interval to delivery was observed to be markedly shorter than in that experienced by the oxytocin group. A relatively high positioning of the fetal head in multiparous women may place insufficient pressure on the uterine cervix in the oxytocin group. The imitating effect of the fetal head provided by the balloon catheter may be the cause of this difference. In studies comparing cervical ripening balloons with dinoprostone (known as a good cervical ripening agent), the mean time interval from induction to delivery was found to be similar between groups (12,13,14,15). Pressure on the uterine cervix may be the major factor providing similar cervical changes with dinoprostone. Interestingly, in our study, the time interval to delivery in primiparous women was similar between both groups. Engagement of the fetal head with the pelvis may the determining factor affecting cervical ripening and the time interval to delivery. Some studies have argued that cervical ripening balloons could impede fetal head engagement with the birth canal and risk cord prolapse (16,17). Other recent studies found that cervical ripening balloons function like a pillow for the fetal head, owing to the ellipsoid and cylindrical nature of the balloon (18,19). In accordance with this idea, caesarean ratios were detected as being similar in both groups.

In a review published in 2008, increased maternal and fetal infection rates were reported as the result of balloon catheter use in labor induction (20). On the other hand, more recent studies found no risk of increased infection due to balloon catheter use (21,22). The results of the present study support these latter studies.

Perception of pain is one of the troublesome adverse effects of catheter insertion. Contrary to our expectations, pain perception in the balloon group was lower as compared with the oxytocin group. There can be several factors affecting this result; perhaps the lack of a need for traction with double- balloon catheter use or the physiologic secretion of oxytocin ensured tolerable pain during labor. However, as the balloon application is a newly preferred method for inducing labor, more frequent patient contact could be achieved as a result of the desire to manage possible problems that might hinder the induction process. This increased attention can cause bias in patients such that they felt more comfortable and safer as compared with those in the oxytocin group.

In the present study, our priority was to compare easily obtained, preserved, applied, and cost-effective methods for the IOL in unfavorable cervices. Dinoprostone could be a good alternative for the study design, as it involves simplicity in application, but its high cost and the difficulties involved in its preservation ruled out its use in this study. However, misoprostol is a good alternative for the study design because it provides cost effectiveness, ease of application, and preservation conditions. Unfortunately, misoprostol is not licensed for use in labor induction in our country. Another limitation of the study was our relative inexperience with the balloon in comparison with oxytocin for labor induction.

In summary, the argument regarding labor induction in unfavorable cervices is still continuing. The aim of the this study was to compare our routine labor induction procedure (oxytocin) with the new cervical ripening balloon catheters in women with unfavorable cervices. Similar cesarean ratios were observed in both groups. The balloon catheters provided a shorter time interval to delivery and lower pain perception during labor, especially in multiparous women. Post-partum hemorrhage, APGAR scores, and fetal and maternal outcomes were similar in both groups.

Oxytocin is still a safe, effective, easily applicable, easily preserved, and cost-effective method for labor induction. However, the results of our study are promising for balloon use, especially in multiparous women. The main problems with cervical ripening balloons are the involved cost and application difficulties. By manufacturing reusable catheters, the cost problem can be solved; application difficulties can be resolved by increasing application frequency. The latter situation is bound up with cost effectiveness. Once these disadvantages are resolved, cervical ripening balloons may become a good alternative to oxytocin for inducing labor, especially in multiparous women. It would be beneficial to support these data with large-scale population-based studies.

## Figures and Tables

**Table 1:**
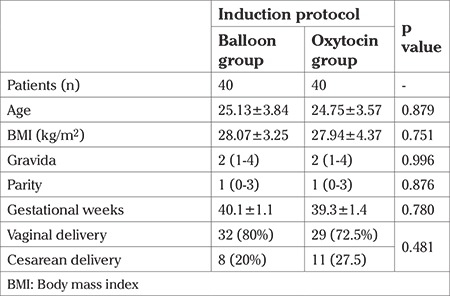
Patient characteristics descriptive statistics were performed. The mean and median values are demonstrated. Standard deviation is marked with ± for the mean values. Minimum and maximum values were demonstrated for the median values with parenthesis

**Table 2:**
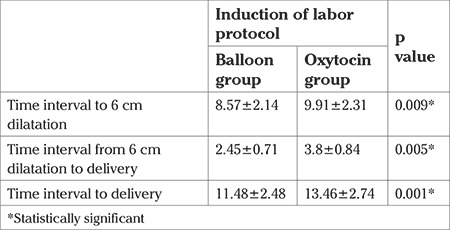
Mean time interval to delivery. Subgroup analyses were performed to observe time interval of latent phase and active phase. The results were statistically significant

**Table 3:**
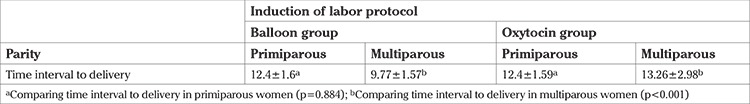
Time interval to delivery according to parity

**Table 4:**
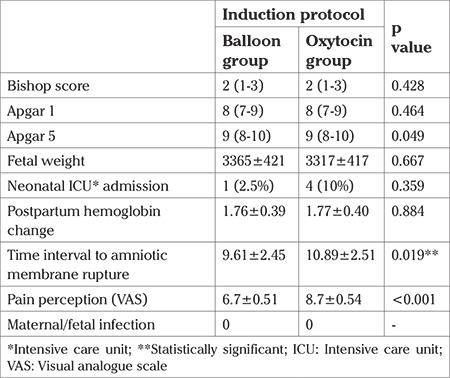
Statistical analysis was established using Students-t test. The mean and median values are demonstrated. Standard deviation is marked with ± for the mean values. Minimum and maximum values are demonstrated for the median values with parenthesis
